# The role of spatiotemporal variation in resources in the diverse movement strategies of temperate ungulates

**DOI:** 10.1098/rspb.2025.1973

**Published:** 2025-11-26

**Authors:** Justine A. Becker, Anna C. Ortega, Jeffrey Beck, Clay B. Buchanan, Thomas Bills, L. Embere Hall, Jacob D. Hennig, Pat Hnilicka, Katey Huggler, Matthew Kauffman, Arthur Middleton, Tony W. Mong, Kevin L. Monteith, Adele Reinking, Hall Sawyer, John Scasta, Brandon M. Scurlock, Jerod A. Merkle

**Affiliations:** ^1^ Department of Ecology, Montana State University, Bozeman, MT, USA; ^2^ Western Wildlife Research Collective LLC, USA; ^3^ Department of Ecosystem Science and Management, University of Wyoming, Laramie, WY, USA; ^4^ Buffalo Field Office, US Bureau of Land Management, Buffalo, WY, USA; ^5^ Wyoming Game and Fish Department, Laramie, WY, USA; ^6^ School of Natural Resources & Environment, University of Arizona, Tucson, AZ, USA; ^7^ US Fish and Wildlife Service, USA; ^8^ Haub School of Environment and Natural Resources, University of Wyoming, Laramie, WY, USA; ^9^ University of Montana, Montana Cooperative Wildlife Research Unit, Missoula, MT, USA; ^10^ Department of Zoology and Physiology, University of Wyoming, Laramie, WY, USA; ^11^ US Geological Survey, Wyoming Cooperative Fish and Wildlife Research Unit, Department of Zoology and Physiology, University of Wyoming, Laramie, WY, USA; ^12^ Department of Environmental Science, Policy, and Management, University of California Berkeley, Berkeley, CA, USA; ^13^ Wyoming Game and Fish Department, Cody, WY, USA; ^14^ Wyoming Cooperative Fish and Wildlife Research Unit, Laramie, WY, USA; ^15^ Cooperative Institute for Research in the Atmosphere, Colorado State University, Fort Collins, CO, USA; ^16^ Graduate Degree Program in Ecology, Department of Fish, Wildlife, and Conservation Biology, Colorado State University, Fort Collins, CO, USA; ^17^ Western Ecosystems Technology, Inc., Laramie, WY, USA; ^18^ Wyoming Game and Fish Department, Pinedale, WY, USA

**Keywords:** animal movement, migration, nomadism, residency, spatiotemporal heterogeneity, ungulates

## Abstract

Animal movement strategies are thought to be determined by the spatiotemporal variation of resources in an environment. Observations of various species indicate that the occurrence of migratory versus resident movements depends on resource predictability and the associated costs and benefits of tracking resource availability versus remaining in a familiar range. Here, we use 21 years of GPS data from seven populations (*n* = 239) of pronghorn (*Antilocapra americana*) and 12 populations (*n* = 283) of elk (*Cervus canadensis*) across Wyoming, USA to test if resource-based hypotheses predict individual movement strategies within a common geographic range. We identified three distinct movement strategies in each species—residents, dual-range migrants and multi-range migrants. Spatiotemporal variation in resources did explain variation in strategies in both pronghorn and elk, with residents experiencing less spatial and greater year-to-year variation than migratory individuals. However, spatiotemporal variation did not predict differentiation between dual- and multi-range migrants in either species. Climatic conditions were also important, especially in elk, where individuals were less likely to be resident when they experienced worse winters. Our findings demonstrate that the movement strategies of temperate ungulates are consistently linked to spatiotemporal resource variation across scales, but additional mechanisms can also facilitate localized behavioural differences.

## 1. Introduction

The ability to move allows animals to buffer themselves against local environmental changes and maintain access to resources that fluctuate across both time and space [1,2]. At large scales, animal movement patterns have been conceptualized into three broad strategies: range residency, migration and nomadism [1]. Residents typically remain in the same location year-round, occupying a small proportion of the overall population range (e.g. urban coyotes (*Canis latrans*) [3]). Migrants exhibit regular, repeated movements between ranges, often tracking seasonal resource availability (e.g. the autumn and spring migrations of many dabbling ducks in North America [4]). Nomads use neither of these strategies and tend to have unpredictable patterns of movement across space (e.g. loggerhead sea turtles (*Caretta caretta*) in the oceanic stage [5]). The movement strategies that individuals employ influence the spatial distributions of populations [1], contribute to the structure and functioning of ecosystems [2,6], and are essential to the effective implementation of conservation efforts [7,8].

**Figure 1.**
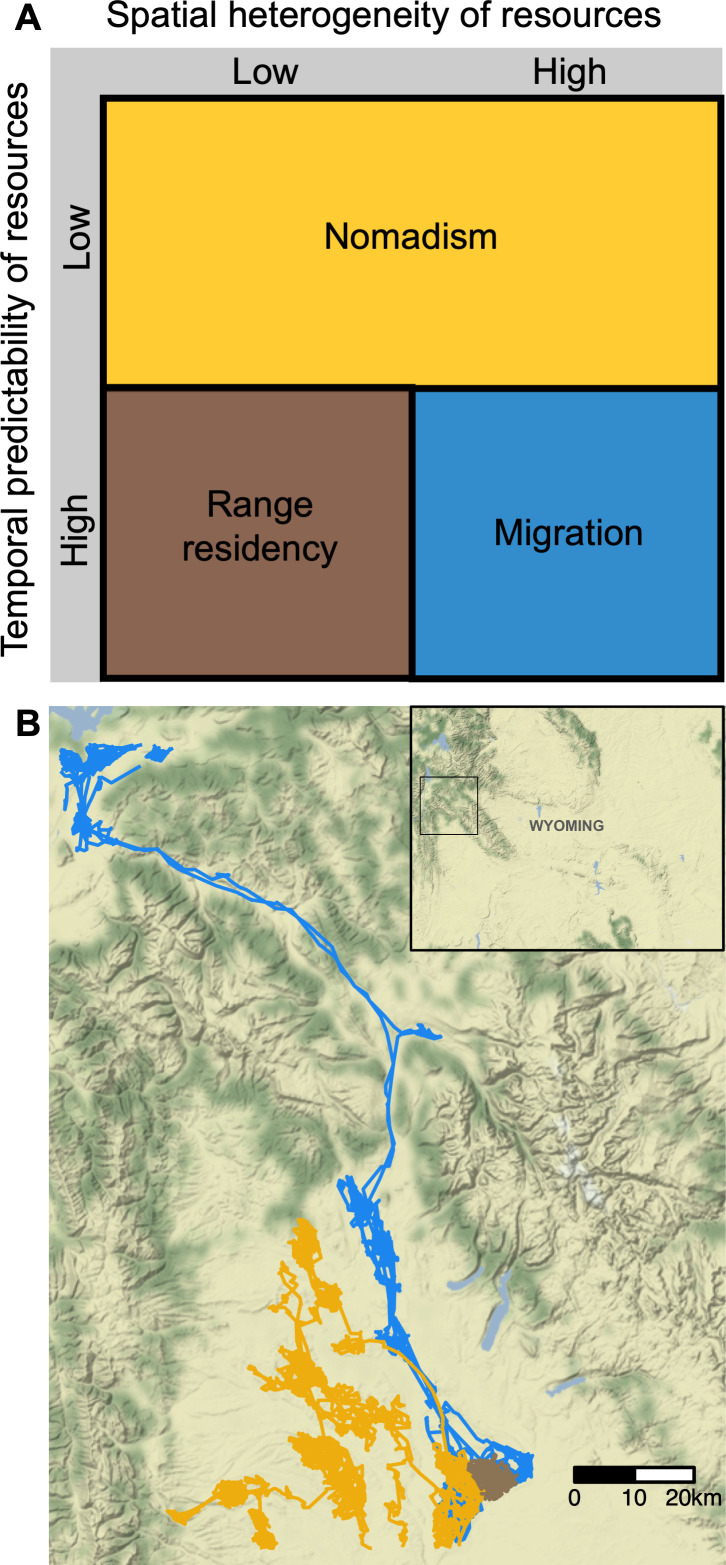
(A) Ecological theory on animal movement strategies predicts that there are specific environmental characteristics that facilitate the evolution of different movement strategies. Figure adapted from [2]. (B) Example tracks of migrant (blue), nomadic (yellow) and resident (brown) pronghorn in the same population in Wyoming, USA (approximate location inset).

The existing ecological framework for understanding animal movement strategies posits that residency, migration, and nomadism should occur in distinct environments, each with specific configurations of spatiotemporal resource availability and distribution [1,9] (figure 1). Migration is expected to develop as a behavioural strategy when resources are spatially heterogeneous, such as the mountains and plains in western North America, but highly predictable in time, such as seasonal environments [1]. Nomadism, on the other hand, is predicted to arise when the temporal predictability of resources is low, such as in desert or steppe biomes with sporadic rainfall [2]. Finally, range residency, in which animals form a static home range or territory, should occur when resources are predictable in space and time and sufficiently abundant across an individual’s range (e.g. tropical primates such as howler (*Alouatta palliata*) and spider monkeys (*Ateles geoffroyi*) feeding on plant foods [1,10]).

Empirical support for the resource-based framework for the evolution of animal movement strategies has been identified across taxa and ecosystems. In ungulates, for example, the classically nomadic saiga (*Saiga tatarica*) inhabit the Eurasian steppe where precipitation is highly unpredictable across the landscape [2,8]. In contrast, in the mountains and plains of North America, where there is high spatial and strong seasonal variation in resource availability between high and low elevations, ungulates such as mule deer (*Odocoileus hemionus*) often exhibit long-distance, seasonal migrations [11,12]. Large-scale studies show that resource waves that move across the landscape over time predict the occurrence of migration in ungulates [13], while residents tend to occur in areas with less spatial variation in resources [14]. Similar patterns have been observed in avian taxa; for example, banded stilt (*Cladorhynchus leucocephalus*) [15] and white ibis (*Eudocimus albus*) [16] exhibit nomadism in response to the varied availability of high-quality habitat, while migrations of Arctic terns (*Sterna paradisaea*) are facilitated by spatiotemporal variation in resource availability [17].

While the increased availability and longevity of fine-scale animal location data has facilitated greater understanding of movement patterns over large spatial and temporal scales, it has also revealed behaviours that appear to contradict resource-based hypotheses for animal movement strategies. Specifically, even among individuals in the same population and environment, movement strategies can vary extensively [18–20]. The phenomenon of partial migration, in which only a proportion of a given population migrates and others remain resident [21], has been well documented in birds, fishes, and ungulates [22–24]. The basic premise of partial migration is that each strategy should be equally successful (i.e. individuals have similar fitness and sub-populations achieve similar intrinsic rates of increase) at certain relative proportions or frequencies within a population [23]. Other studies have identified a range of taxa in which a proportion of a largely migratory or resident population appears to exhibit patterns more akin to nomadic movement [2]. In both cases, the movement strategies used by a given species can also vary from one population to the next (e.g. [25,26]).

The relationship between animal movement strategies and spatiotemporal variation in resources is less well understood when several distinct movement strategies occur in the same species in the same geographic region. In these circumstances, it is possible that resource-based hypotheses are still broadly applicable, but some landscapes may have enough variability in habitat configuration within a population’s range to support multiple strategies. Some landscapes may also abruptly transition between patterns of high and low spatiotemporal variation over small spatial scales (e.g. where homogeneous grassland and heterogeneous montane habitats meet; the centre point of figure 1A), in which case some apparent overlap among different strategies is expected. In both circumstances, the predicted outcome would include multiple strategies where the proportion of each strategy reflects the relative benefits of each strategy at low population densities [23,27,28]. Empirically testing these ideas is challenging, however, because we can only observe the realized resource environment that each individual inhabits given their chosen movement strategy. It is important to also consider the possibility that the association between a given movement strategy and spatiotemporal variation in resources could arise, in part, from the movement strategy itself exposing individuals to different resource environments. For example, an individual who migrates between seasonal ranges in a mountainous environment would experience higher spatial and lower year-to-year variation in resources compared to residents.

If no evidence for a relationship between movement patterns and spatiotemporal resource distribution among individuals living in close proximity is detected, there are two primary alternative hypotheses. First, each strategy may be successful at least some of the time or for some individuals and is maintained in localized portions of a population via social learning and cultural transmission [29]. For instance, different proportions of a population may specialize in exploiting alternative resource niches (e.g. [30]) and pass their specific movement strategies down to the next generation by learning from parents or other conspecifics. Second, other natural and anthropogenic environmental conditions, such as climate, landscape connectivity, agriculture, development and hunting pressure, may play a greater role in observed movement strategies when differentiation among individuals occurs at a fine scale. Among these factors, there is strong evidence to suggest that snow accumulation and winter severity can influence the movements of both resident and migratory ungulates either by acting as a barrier to movement or by increasing movement rates or prompting range shifts to escape adverse winter conditions [31–34]. Croplands and proximity to human development have also been shown to affect how ungulates move across the landscape and may serve as attractive features due to the provision of supplementary food sources or result in avoidance to reduce negative encounters with humans [29,30,34–37].

Here, we used a multi-species comparative approach with two ungulate species that are sympatric but have different ecological requirements and life histories to test resource-based hypotheses for individual movement strategies. We used GPS-collar data from seven populations of pronghorn (*Antilocapra americana*) and 12 populations of elk (*Cervus canadensis*) across the core of their North American distribution (Wyoming, USA). In this region, anecdotal evidence suggests that resident, migrant, and nomadic individuals of both pronghorn and elk live in close proximity in the same populations [23,25,32,38]. We tested the following objectives: *objective 1*: quantify individual movement strategies (i.e. is there quantitative evidence of residency, migration, and nomadism in the same populations, and in what relative proportions?) in pronghorn and elk across our study area; *objective 2*: test the theoretical predictions from the hypothesis that animal movement strategies are determined by the spatiotemporal variation of resources in an environment (as outlined by Mueller & Fagan [1] and shown in figure 1A) for two species in a single region, i.e. residents are found in areas of low spatial and high year-to-year variation, and vice versa for migrants, while nomads are associated with areas of high year-to-year variation (low temporal predictability); and *objective 3*: test alternative hypotheses related to climatic conditions and anthropogenic features that may explain the observed variability in pronghorn and elk movement strategies. We focused on climatic conditions and anthropogenic features because of the existing evidence that supports a relationship with pronghorn and elk movement [31–34]. Specifically, we hypothesized that the following factors influence individual movement strategies: snow accumulation and winter severity [31–34] (representing climatic conditions), and the presence of croplands and proximity to human development [29,30,34–37] (representing anthropogenic features). Current evidence suggests that each of these factors can both increase and decrease movement of ungulates; therefore, we did not make specific directional predictions about the relationship between these variables and individual movement strategies [29–37].

## 2. Methods

### (a) Movement data collection and cleaning

We synthesized GPS movement data from *n* = 239 adult female pronghorn and *n* = 283 adult female elk from seven pronghorn and twelve elk populations in Wyoming, USA, collected between 2000 and 2021. We operationally defined a population as all collared animals captured within a similar area such as a state-designated herd management unit. Study populations were distributed across Wyoming, ranging from the mountain foothills in the Upper Green River, Bighorn and Wind River Basins in the north and west, to more open, arid environments in the Red Desert and Shirley Basin in south-central and southeastern Wyoming. Detailed descriptions of population habitats and climatic conditions can be found in [34,39,40] for pronghorn and [37,41–44] for elk, and in electronic supplementary material, appendix S1.

All movement data were collected from GPS collars affixed to individual adult (>1 year old) female pronghorn and elk following capture via helicopter net-gunning. All captures followed protocols in accordance with guidelines from the American Society of Mammalogists [45] and permits (electronic supplementary material, appendix S1). The fix rate of the GPS collars ranged from 1 to 4 h for pronghorn and 1–24 h for elk, with a median of 1 h across all study populations (see electronic supplementary material, appendix S1).

### (b) Objective 1: quantifying individual movement strategies

Two of the most common methods for classifying the movement strategies of animals include using net-squared displacement curves [46] or estimating the spatial overlap between seasonal ranges [47]. While the former approach is particularly effective for distinguishing migratory individuals, its utility is limited for populations that do not conform to typical migratory movement patterns [47]. To determine individual movement strategies in our study populations, we adapted methods for measuring migratory movements using seasonal range overlap from Cagnacci *et al.* [47]. We also tested and found strong agreement between the movement classifications derived from the spatial overlap method and the net-squared displacement approach (electronic supplementary material, appendix S2). We estimated the extent of overlap in individual space use or ranges over different periods and used these measurements of spatial overlap as continuous metrics to quantify alternative movement strategies. Our final dataset only included individuals for which we had at least 21–23 months of consecutive data (the temporal extent depended on the time of year that each individual was collared initially and the seasonal cycles; see electronic supplementary material, appendix S1) such that we could compare space use both within and across years. We generated a series of occurrence distributions (ODs) for each individual using either a Brownian bridge movement model or kernel utilization distribution and a grid resolution of 150 × 150 m^2^ [48,49]. For each individual, we estimated the following ODs: a summer range for each year of available data, a winter range for each year of available data, and individual monthly ranges for each month across the available dataset. Summer was defined as 1 June–31 August and winter was defined as 1 December–28/29 February. We used the calendar month to define the data period for the monthly ODs. This resulted in a minimum of 21–23 distinct monthly ODs for each individual.

We then measured the volumetric overlap, which accounts for relative intensity of use, between pairwise combinations of the ODs for each individual using the 95% ODs and the Bhattacharyya affinity overlap index [49–52]. We distilled the measurements of pairwise OD overlap into four distinct and continuous movement metrics. First, we calculated the average seasonal overlap (metric 1), which we measured as the overlap between summer and winter ranges in the first year and the overlap between summer and winter ranges in the second year. This metric measured the extent to which individuals use distinct ranges between summer and winter and estimated each individual’s tendency towards a resident (higher values nearing 1) or migratory (lower values nearing 0) strategy. Second, we calculated the overlap between summer ranges (metric 2) and the overlap between winter ranges (metric 3) across years. We expected true migratory individuals to use the same seasonal ranges from year to year (higher values towards 1), whereas switching seasonal ranges from year to year (lower values towards 0) could indicate nomadism or another alternative strategy. Finally, we calculated the mean overlap between all pairwise combinations of the monthly ODs that were >1 month apart for each individual, to minimize the effects of temporal autocorrelation (metric 4); for instance, an OD from July would be compared to the ODs in all other months except for June and August. We termed this metric the average annual overlap and expected it to measure each individual’s tendency towards a resident strategy (higher values nearing 1) within a single year. We then used a subset of these movement metrics (i.e. metric 1—average seasonal overlap, metric 2—summer range overlap, metric 3—winter range overlap) and *k*-means clustering [25,53] to classify animals into three distinct movement strategies. We found the following strategies in our study populations: (i) residents, (ii) true migration in which animals consistently used the same seasonal ranges from year to year (i.e. dual-range migrant), and (iii) a more flexible migratory strategy in which animals had different summer or winter ranges from year to year (i.e. multi-range migrant/seasonally nomadic, which has been previously observed and defined in [54,55]; electronic supplementary material, appendix S3, figure S1). Based on anecdotal evidence from our study populations, we had expected to find resident, migrant, and nomadic strategies; however, our quantitative assessment of these herds supported the presence of multi-range migrants rather than true nomads.

### (c) Objective 2: individual movement strategies and spatial and year-to-year variation in resources

To quantify the resource environment experienced by each individual, we estimated their year-round range as a 95% kernel utilization distribution, using 21–23 months of data for each individual, depending on availability, and a grid resolution of 150 × 150 m^2^ [48,49]. We then extracted environmental covariates that are known to be highly correlated with resource availability for ungulates to each individual year-round range (see electronic supplementary material, appendix S1). We estimated spatial variation in resources by calculating the s.d. of each environmental covariate across each individual year-round range. For covariates with multiple years of data, we first calculated the mean value across years for each grid cell within the year-round range and then estimated the s.d. of these mean values. We then used a principal components analysis to aggregate the standard deviations of all environmental covariates (many of which were strongly correlated) into a single measurement of spatial variation in resources (see electronic supplementary material, appendix S1, S3, figures S2 and S3).

We measured temporal predictability of the resource environment as the year-to-year variation in resources within each individual year-round range. We used three variables known to be strongly associated with resource availability for temperate ungulates—date of peak spring green-up, length of the primary growing season (i.e. spring length in days) and total aboveground herbaceous biomass (lbs per acre) [56]. We estimated resource variation between years by extracting the mean value for each covariate in each year across all grid cells in each individual range and then calculated the standard deviation of these annual mean values (electronic supplementary material, appendix S1). Because the date of peak spring green-up and spring length were strongly correlated (*R*^2^ > 0.7), we used generalized linear regression models and a comparison of the Akaike information criterion corrected for small sample sizes (AICc) to identify which of these covariates performed best in explaining variation in individual movement strategies (see electronic supplementary material, appendix S1). We identified the combination of spring length and herbaceous biomass as the top model for both species and used these covariates to represent year-to-year variation in resources (electronic supplementary material, appendix S3, figures S2 and S3).

We used mean daily snow depth to determine overall winter conditions and relative winter severity (see electronic supplementary material, appendix S1), which we used as indices of climatic conditions for each individual’s year-round range [31–34]. We used percent agriculture and mean distance to roads in each individual’s year-round range as indices of anthropogenic features on the landscape (see electronic supplementary material, appendix S1) [34–37].

We fitted generalized linear regression models to assess the relationship between individual movement strategies—as defined by our metrics of spatial overlap—and spatial and year-to-year variation in resources. For each species, we fitted three candidate models for four different subsets of the overall dataset, including (i) all animals, (ii) only dual-range migrants and multi-range migrants, (iii) only residents and dual-range migrants, and (iv) only residents and multi-range migrants. In each model set, we used one of the spatial overlap metrics as the response variable to differentiate between the movement strategies being compared in each model set (e.g. we used seasonal overlap as the response variable for models comparing residents and dual-range migrants).

The three candidate models were the same across each model set and included (i) spatial variation and (ii) year-to-year variation in resources (represented by both spring length and aboveground herbaceous biomass) as predictor variables, and (iii) an intercept-only model (electronic supplementary material, appendix S3, table S3). We did not include spatial and year-to-year variation in resources in the same model because these variables were often highly correlated (*R*^2^ > 0.6). For the model sets with all animals, we used seasonal overlap as the response variable (i.e. high values represented residents, low values represented dual-range or multi-range migrants). For the model sets with dual-range and multi-range migrants, we used overlap among winter ranges across years as the response variable (i.e. high values represented dual-range migrants, low values represented multi-range migrants). To compare residents and dual-range migrants, we also used seasonal overlap as the response variable (i.e. high values represented residents, low values represented dual-range migrants). Lastly, we used annual overlap as the response variable in the model sets with residents and multi-range migrants (i.e. high values represented residents, low values represented multi-range migrants). We used a beta distribution for each model because our response variables were proportional values bounded between 0 and 1 (electronic supplementary material, appendix S1). We scaled and centred all predictor variables within each subset of data prior to model fitting and compared model fits using AICc.

We conducted a sensitivity analysis to verify the robustness of our results by separating the contributions of the resource environment and individual behavioural decisions on the observed relationship between spatial and year-to-year variation in resources and individual movement strategies (as defined by our metrics of spatial overlap). We tested the consistency of this relationship when all individuals had a standardized domain of availability from which we sampled the spatial and year-to-year variation in resources. In contrast to our primary analysis in which the availability domain was determined by each individual’s year-round range derived from GPS location data, this standardized domain was independent of the locations where individuals established their ranges. Thus, it reflects the overall resource environment available to that individual rather than only the environment they experienced while using their movement strategy. We delineated a circular buffer of a standardized radius around the centroid of each individual’s GPS locations in winter (1 December–28/29 February). The buffer radius was approximately equal to the 95% quantile of the areas of the annual ranges estimated for pronghorn (3765 km^2^) and elk (1647 km^2^). We used these standardized domains to re-calculate the spatial and year-to-year variation in resources for each individual, following the same procedure described above for the empirical year-round ranges. We then replicated the same set of beta regression models for objective 2 and compared the results.

### (d) Objective 3: alternative hypothesis testing

We fitted another series of generalized linear models with a beta distribution to test our alternative hypotheses on how climatic conditions and anthropogenic features may influence the individual movement strategies we defined in our study system. We repeated the same data partitioning and model fitting procedures as described for objective 2 but tested seven candidate models for each of the four model sets for each species (electronic supplementary material, appendix S3, table S4). We compared our alternative hypotheses directly against the resource-based hypotheses tested in objective 2 by including the top model from objective 2 for each of the four model sets as one of the seven candidate models. The other six models included an intercept-only model, a model with climatic conditions (overall winter conditions and relative winter severity), a model with anthropogenic features (per cent agricultural lands and distance to roads), and three other models with a combination of these variables and the covariates from the top model in objective 2 (electronic supplementary material, appendix S3, table S4). We selected the best-fitting model using a comparison of AICc.

## 3. Results

### (a) Objective 1: quantifying individual movement strategies

**Figure 2.**
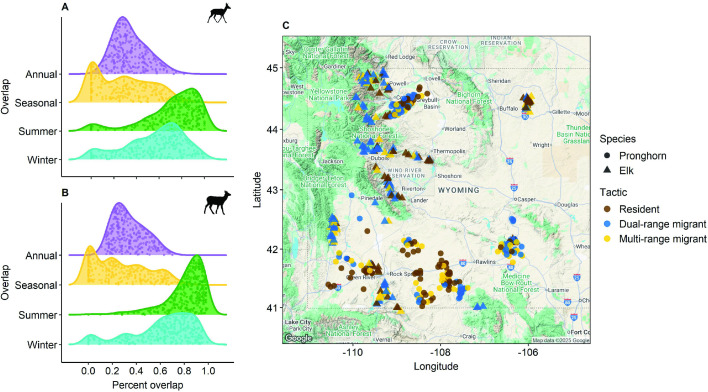
Movement strategies of pronghorn and elk across Wyoming, USA. Probability densities of percent overlap between monthly ranges (i.e. annual overlap; purple), winter and summer ranges (i.e. seasonal overlap; yellow), summer ranges (green) and winter ranges (blue) for *n* = 239 pronghorn (A) and *n* = 283 elk (B). Spatial distribution of *n* = 239 pronghorn across 7 populations and *n* = 283 elk across 12 populations in Wyoming, USA (C). Points represent the centroid location of an animal’s home range and are coloured by movement strategy—residents (brown), dual-range migrants (blue) and multi-range migrants or seasonal nomads (yellow).

Both pronghorn and elk displayed diverse movement strategies (figure 2; electronic supplementary material, appendix S3, tables S1, S2 and S4–S6). Average seasonal overlap was skewed towards 0 with a median of 0.27 (range: 0–0.91) for pronghorn and 0.23 (range: 0–0.84) for elk (figure 2; electronic supplementary material, appendix S3, tables S1, S2 and S4–S6). The average summer overlap and average winter overlap from one year to the next was relatively high, with a median of 0.86 for summer and 0.68 for winter for elk, and a median of 0.78 for summer and 0.62 for winter for pronghorn (figure 2). Average annual overlap was consistent across species, with a median overlap of 0.31 for both pronghorn and elk and ranges of 0.07–0.82 and 0.11–0.70, respectively (figure 2; electronic supplementary material, appendix S3, figures S5 and S6).

The *k*-means clustering identified approximately 38% of pronghorn and 33% of elk as residents, with high overlap in space use year-round and across years. A similar proportion of individuals (38% of pronghorn and 45% of elk) were identified as dual-range migrants, with distinct summer and winter ranges that were consistent across years. The remaining 24% of pronghorn and 22% of elk shared a similar movement strategy; however, this strategy diverged from the traditional definition of nomadism in which individuals should move through the landscape in a seemingly unpredictable way [2]. These individuals instead displayed multi-range migration [54,55] or seasonal nomadism [2] characterized by low seasonal overlap and low overlap between winter ranges across years. A few pronghorn in this group also had low overlap between summer ranges, which suggests a movement pattern closer to true nomadism, but we did not analyse this movement strategy separately because of the small sample size. Likewise, a few resident pronghorn (*n* = 5) and dual-range migrant elk (*n* = 4) had noticeably lower summer range overlap (<25%) compared to other individuals in their respective groups.

### (b) Objective 2: individual movement strategies and spatial and year-to-year variation in resources

**Figure 3.**
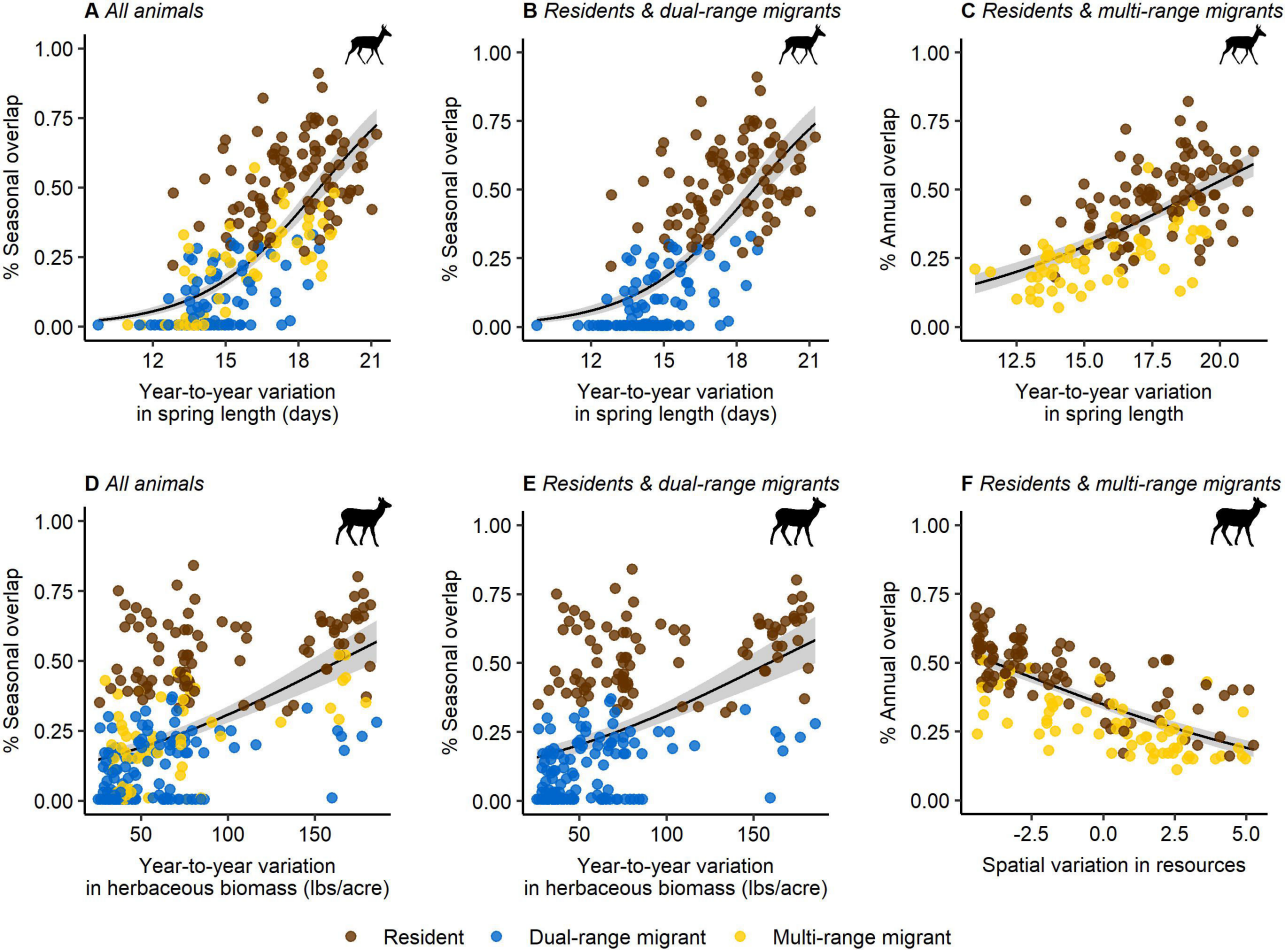
Seasonal and annual overlap in space use as a function of spatial variation in resources and year-to-year variation in plant phenology (i.e. spring length (days), aboveground herbaceous biomass (lbs per acre)) for pronghorn (A–C) and elk (D–F). The colour of points represents the movement strategy for each individual—residents (brown), dual-range migrants (blue) and multi-range migrants (yellow). Pronghorn and elk exhibited resident strategies when spring length and herbaceous biomass were less predictable across time. Black lines represent the prediction of the coefficient estimate from a beta regression model. Grey bands represent the 95% confidence interval.

In almost all cases, year-to-year variation in resources (the consistency of spring length and/or aboveground herbaceous biomass) was the strongest predictor of pronghorn and elk movement strategies (electronic supplementary material, appendix S3, tables S5–S8). Spatial variation (the aggregate variation in all environmental covariates across an individual’s range) in resources was also a significant predictor of individual movement strategies but was only the top model in one model set (electronic supplementary material, appendix S3, tables S5–S8 and figure S7). In both species, we found strong evidence that individuals were more likely to be residents (i.e. high overlap between seasonal ranges) in areas with high year-to-year variation in resources and more likely to be dual-range migrants (i.e. low overlap between seasonal ranges) in areas with more predictable resources (figure 3). These relationships were consistent when we considered all animals (*p*s*–R*^2^ = 0.52 for pronghorn, *p*s*–R*^2^ = 0.27 for elk), and only those individuals identified as either residents or dual-range migrants (*p*s*–R*^2^ = 0.51 for pronghorn, *p*s*–R*^2^ = 0.26 for elk; figure 3A,B,D,E; electronic supplementary material, appendix S3, tables S7 and S8).

We also found strong evidence that the divergence between the resident and multi-range migrant strategies was associated with either spatial or year-to-year variation in resources. In pronghorn, individuals were more likely to be resident (i.e. high annual overlap in space use) when there was more variation in spring length across years (*p*s*–R*^2^ = 0.42, *β* = 0.46 [0.37–0.55]; figure 3C; electronic supplementary material, appendix S3, table S7). In elk, individuals were more likely to be resident in areas with low spatial variation in resources (*p*s*–R*^2^ = 0.52, *β* = −0.48 [−0.55 to −0.40]; figure 3F; electronic supplementary material, appendix S3, table S8).

We did not find strong evidence that spatial and year-to-year variation in resources explained the divergence between dual-range migrant and multi-range migrant strategies in either species. For elk, our model selection identified the null (intercept only) model as the top model (electronic supplementary material, appendix S3, table S8). For pronghorn, individuals were more likely to be dual-range migrants compared to multi-range migrants (i.e. higher overlap between winter ranges across years) when year-to-year variation in aboveground herbaceous biomass was higher (*β* = 0.33 [0.15–0.50]; electronic supplementary material, appendix S3, table S7 and figure S8B). However, there was also weak evidence (*β* = −0.18 [−0.35 to −0.01]; electronic supplementary material, appendix S3, table S7) that individuals were more likely to be dual-range migrants when year-to-year variation in spring length was lower and the explanatory power of this model was relatively low (*p*s*–R*^2^ = 0.11; electronic supplementary material, appendix S3, tables S7 and figure S8A).

The relationship between spatial and year-to-year variation in resources and individual movement strategies remained consistent between the models based on empirical year-round ranges and the models based on a standardized domain of availability. The only difference in the model selection process was that the top model for the dual- and multi-range migrant dataset for pronghorn was no longer the model that included year-to-year variation in spring length and aboveground herbaceous biomass, but the model that included only spatial variation in resources as a covariate (electronic supplementary material, appendix S3, table S9). This model revealed a positive relationship between spatial variation in resources and overlap between winter ranges across years, demonstrating that these individuals were more likely to be dual-range migrants compared to multi-range migrants in areas with more spatial variation in resources (*p*s*–R*^2^ = 0.24, *β* = 0.62 [0.45, 0.79]; electronic supplementary material, appendix S3, table S11). All other top models remained the same between the analyses based on empirical year-round ranges and those based on the standardized domain of availability (electronic supplementary material, appendix S3, tables S9 and S10).

We found some changes in effect size and explanatory power using the standardized domain of availability, but this varied between species. For elk, the *p*s*–R*^2^ values and coefficient estimates for the significant covariates remained similar between the sensitivity analysis and our primary analyses using the empirical year-round ranges, apart from a lower *p*s*–R*^2^ value for the resident and multi-range migrant model (0.36 compared to 0.52) (electronic supplementary material, appendix S3, tables S11 and S12). For pronghorn, we observed slightly lower effect sizes for the significant covariates that were present in models across both analyses, and the *p*s*–R*^2^ values were roughly half of those same values in the primary analyses (apart from the model set for which the top model changed and the *p*s*–R*^2^ value actually increased—dual- and multi-range migrants) (electronic supplementary material, appendix S3, tables S11 and S12).

### (c) Objective 3: alternative hypothesis testing

**Figure 4.**
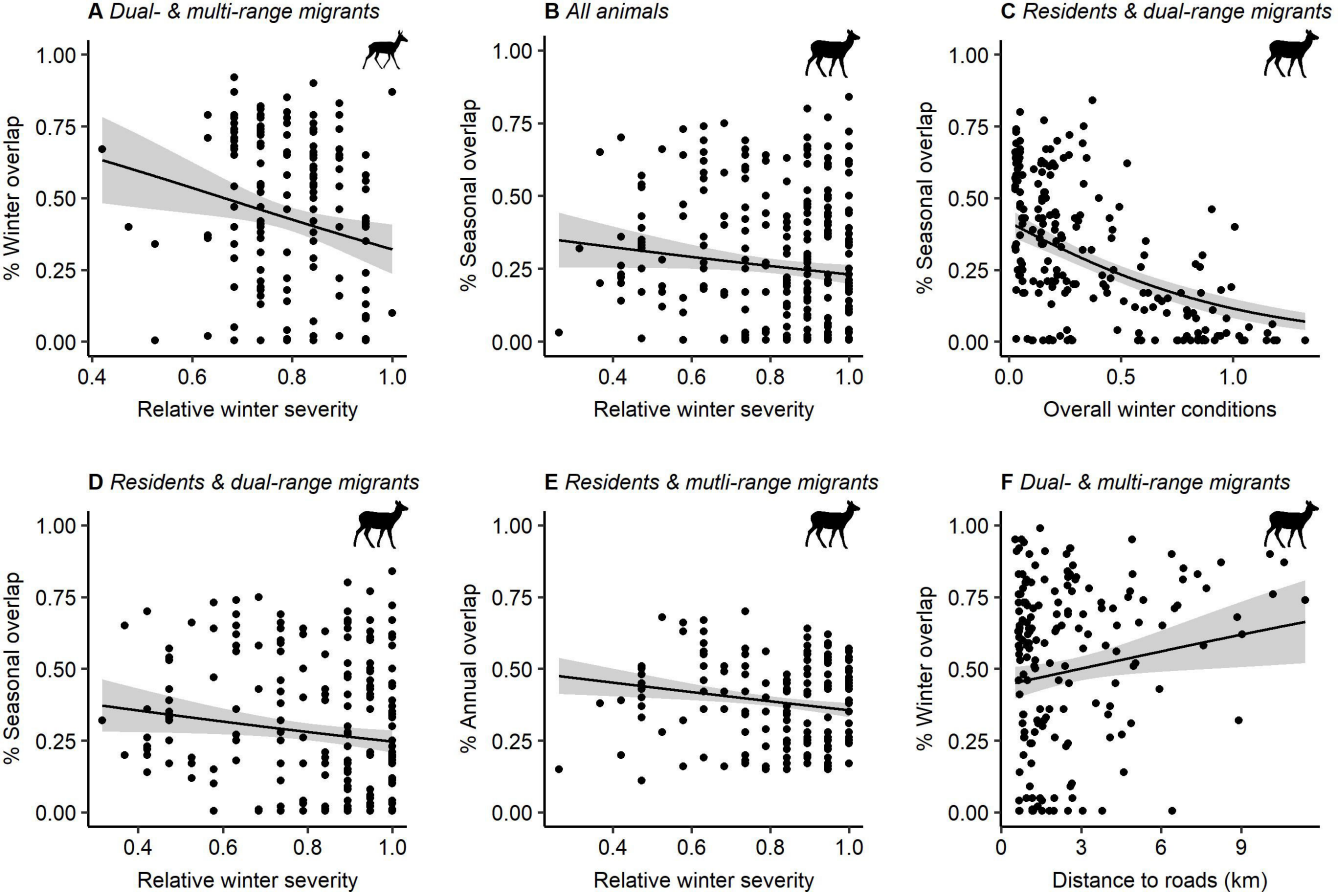
Significant effects of alternative environmental variables on movement strategies for pronghorn and elk. Pronghorn exhibited multi-range migration behaviour during winter (i.e. lower overlap between winter ranges) when winters were relatively more severe (A). Elk exhibited more mobile behaviour (i.e. dual-range migrants or multi-range migrant strategies represented by lower seasonal and annual overlap) when winters were more severe and overall winter conditions were worse (higher values) (B–E) and exhibited multi-range migrant behaviour during winter when they were closer to roads (F). Black lines represent the prediction of the coefficient estimate from a beta regression model. Grey bands represent the 95% confidence interval.

In testing our alternative hypotheses—climatic conditions and anthropogenic features—for individual movement strategies, we found continued support for year-to-year variation in resources as an important predictor of movement strategies in pronghorn (electronic supplementary material, appendix S3, tables S13 and S15). For the model sets that included all animals, only residents and dual-range migrants, and only residents and multi-range migrants, the top model remained the same as the top model identified in objective 2 (electronic supplementary material, appendix S3, tables S13 and S15). For the model set that included only dual- and multi-range migrants, we found that year-to-year variation in spring length (*β* = −0.36 [−0.56 to −0.16]) and aboveground herbaceous biomass (*β* = 0.34 [0.14–0.54]) had similar relationships with the dual- and multi-range migrant strategies as identified in objective 2 (electronic supplementary material, appendix S3, tables S15 and figure S8), but the top model also included relative winter severity, per cent agricultural lands, and distance to roads, and had a higher *p*s*–R*^2^ (0.16 compared to 0.11; electronic supplementary material, appendix S3, tables S13 and S15). Of these covariates, only relative winter severity was significantly associated with whether an individual was a dual- or a multi-range migrant—individuals were more likely to be a multi-range migrant (i.e. lower overlap between winter ranges across years) when winter severity was higher (*β* = −0.24 [−0.42 to −0.06]; figure 4A; electronic supplementary material, appendix S3, table S15).

In elk, we found that climatic conditions and anthropogenic features were predictors of individual movement strategies, and in several cases, better predictors than spatial or year-to-year variation in resources (figure 4B–F). For all model sets, the top model contained at least one climatic or anthropogenic variable, and three of the four model sets contained either overall winter conditions or relative winter severity (electronic supplementary material, appendix S3, tables S14 and S16). When present in the top model, spatial and year-to-year variation in resources retained the same relationships with individual movement strategies as identified in the models for objective 2 (electronic supplementary material, appendix S3, table S16). Across all elk, we found some evidence to suggest that individuals had lower overlap between seasonal ranges with higher relative winter severity (*β* = −0.14 [−0.26 to −0.01]; figure 4B; electronic supplementary material, appendix S3, table S16), but year-to-year variation in spring length and aboveground herbaceous biomass was retained in the top model and the latter had the greatest effect size (*β* = 0.51 [0.39–0.63]; electronic supplementary material, appendix S3, tables S14 and S16). For those elk identified as being either a dual- or a multi-range migrant, we found only weak evidence (*p*s*–R*^2^ = 0.04) that the presence of anthropogenic features influenced individual movement strategies. These elk were more likely to be multi-range migrants (i.e. lower overlap between winter ranges across years) when nearer to roads (*β* = 0.19 [0.02–0.35]; figure 4F; electronic supplementary material, appendix S3, table S16). For those elk identified as resident or dual-range migrants, we found that climatic conditions were a better predictor of individual movement strategies than spatial and year-to-year variation in resources (*p*s*–R*^2^ = 0.30; electronic supplementary material, appendix S3, tables S14 and S16). Both relative winter severity (*β* = −0.16 [−0.29 to −0.02]; figure 4D; electronic supplementary material, appendix S3, table S16) and overall winter conditions *(β* = −0.57 [−0.71 to −0.42]; figure 4C; electronic supplementary material, appendix S3, table S16) were negatively correlated with seasonal overlap, indicating that individuals were more likely to be resident in areas with more mild winters. For those elk identified as being either a resident or multi-range migrant, spatial variation in resources remained an important predictor of individual movement strategies (*β* = −0.50 [−0.57 to −0.42]; electronic supplementary material, appendix S3, table S16); while relative winter severity was also in the top model (*β* = −0.11 [−0.19 to −0.04]; figure 4E; electronic supplementary material, appendix S3, table S16), the addition of this covariate resulted in only a marginal improvement in explanatory power (*p*s*–R*^2^ = 0.54 compared to 0.52 in the top model for objective 2; electronic supplementary material, appendix S3, tables S14 and S16).

## 4. Discussion

We used long-term GPS-location data from two ungulate species with varied life histories to test resource-based hypotheses for individual movement tactics when several movement strategies co-exist within a common geographic range. We found that individual movement strategies varied widely and that spatial and year-to-year variation in resources—ranging from environments with high spatial variation and predictability in resource availability to more spatially homogeneous and less predictable regions—are important factors underpinning localized differences in movement strategies. We identified three distinct movement strategies in each species—residents and dual-range migrants were present as expected, but the third strategy did not conform to traditional nomadic movements. Instead, these individuals were multi-range migrants who adopted semi-nomadic movement patterns but only seasonally. In both species, we found that year-to-year variation in resources explained variation (52% in pronghorn and 27% in elk) among individuals using either resident or migratory (both dual- and multi-range migrants) movement strategies: resident individuals were consistently found in areas with less spatial and greater year-to-year variation. These relationships held when we isolated the effects of the resource environment on movement using a standardized domain of availability. However, we found weak support in pronghorn and no support in elk that spatial and year-to-year variation in resources drives differentiation between dual-range migrants and multi-range migrants. Climatic conditions were also important for individual movement strategies, and primarily in elk—individuals were less likely to be resident when they experienced worse winter conditions or relative winter severity. Overall, we demonstrate that resource variation remains a key component of ungulate movement strategies at fine scales, but that factors such as climatic conditions also play a role in shaping diverse movement strategies in these species.

Our results support the well-known idea that the benefits of migration, and therefore its occurrence, will be greatest in landscapes with cyclic patterns of availability in resources that also vary in space [1,13]. In both species, residents tended to occur in areas with low temporal predictability (e.g. spring length was more variable from year to year) and low spatial variation (e.g. high prairie/grassland habitats), while migrants occupied regions where resource availability was predictable from year to year but varied in space. Recent explorations of animal migrations have shown that many of the benefits of seasonal movements for herbivores are derived from the migration path itself as animals ‘surf the green wave’ and optimize nutritional gains en route [56–58]. While the phenology of the green wave shapes migration timing, length and dynamics [13,59], we found that migration was best predicted by lower year-to-year variation in the length of spring or the amount of aboveground herbaceous biomass. This finding suggests that consistency in the amount of resources available is important for understanding the occurrence of migratory strategies and supports recent work that found a similar pattern for pronghorn across Montana, USA [60].

Despite anecdotal evidence of nomadism in our study populations, other than a handful of pronghorn individuals, we did not find quantitative support for this movement strategy. In both species, we instead identified an alternative movement strategy that did not fit the definition of residency or dual-range migration, in which 22–24% of individuals tended to move between summer and winter ranges but switched seasonal ranges year to year (i.e. multi-range migrants or seasonal nomads). While we did have multiple years (21–23 months) of GPS-location data for each individual, the identification of true nomadic movements may require even longer time frames, particularly for relatively long-lived species. Ecologically, this finding indicates that some behavioural flexibility in movement decisions may be beneficial for animals in environments where seasonal variation can result in extreme and challenging conditions that may be less predictable from year to year. Generally, we found multi-range migrants to be associated with environments with more severe winters, which echoes previous research suggesting that this strategy is used on a facultative basis to escape adverse conditions [19,32]. The multi-range migrant strategy has been identified across a variety of temperate ungulate species, including other populations of elk [61] and pronghorn [32], as well as moose [19] and mule deer [54]. Our results from >20 populations highlight the widespread and common nature of this behaviour.

The qualitative agreement between the empirical-range models and the standardized domain models supports a consistently significant effect of spatial and year-to-year variation in resources on the movement strategies of pronghorn and elk. We found lower effect sizes and *p*s*–R*^2^ values in a majority of cases (although the lowest *p*s*–R*^2^ for a non-null model was still >0.2). This difference was largely unsurprising because, in addition to standardizing the domain of availability, these ranges were also standardized to a circular distribution, which likely reduced the environmental variation within each individual’s habitat domain relative to their empirical range. For example, many of the individuals in our dataset with high spatial variation in their observed ranges have narrow, elongated distributions because they migrate every year from low-elevation winter ranges to high-elevation summer ranges. The difference in explanatory power also indicates that there is likely a positive feedback loop between the resource environment and individual behavioural decisions. For example, short-distance migrations could arise in environments with some degree of spatial and year-to-year variation in resources and slowly extend over greater distances to exploit larger gradients of spatiotemporal variation as individuals gain more information about their environment [1,2].

Our findings also emphasize the importance of climatic conditions for informing individual movement choices. Both elk and pronghorn are known to exhibit dynamic responses to winter conditions [23,25,32,37,62–65]. The divergence between residents and dual-range or multi-range migrants in elk was best explained by models that included the winter conditions each individual experienced. Individuals who experienced worse winters had lower seasonal overlap and were more likely to be dual-range or multi-range migrants compared to residents. We did not find the same importance of winter conditions for pronghorn; spatial and year-to-year variation remained the most important predictors of seasonal range overlap. This difference may be due to the range of environmental conditions experienced by our study populations. Elk in our study could be coarsely sorted into two groups—those living in more homogeneous, low snow areas (plains regions) and those living in mixed elevation, high snow areas (montane regions). The stronger relationship between movement strategies and winter for elk likely emerged from the contrast in environmental conditions experienced by each of these groups and the sensitivity of ungulate movements to winter dynamics [66,67]. Pronghorns resided in comparatively similar environmental conditions, in plains regions with less snow, and thus their movement patterns may be more heavily influenced by other landscape features, namely spatial and year-to-year variation in resources [56–58,68,69].

We did not find a strong effect of anthropogenic features on elk or pronghorn movement strategies. Other research has demonstrated that pronghorn and elk movements can be heavily impacted by anthropogenic activities such as roads, fences and energy development [26,34–36,70]. Our use of relatively coarse metrics that could be readily and reliably estimated across the large spatial extent of our study area to index human activities (i.e. distance to roads and percent agricultural lands) may have obscured some of the underlying relationships. Assessing the same relationships examined here but with metrics more tailored to addressing specific questions about anthropogenic effects on individual movement strategies would be an interesting avenue for further research. For example, there is increasing evidence that sociality is an essential component of animal movement decisions, particularly with respect to navigating anthropogenically modified landscapes [71–73], and research in other species such as red deer (*Cervus elaphus*) [74] and moose [19] has found that migratory and nomadic movements can be prompted by the onset of the hunting season.

Recent declines in many migratory and nomadic species have resulted in a strong push to understand and conserve these movements [2,12,75–77]. We empirically tested the role of resource variation for animal movement strategies in pronghorn and elk herds where several movement strategies were all found within the same geographic region in Wyoming, USA. Our results emphasize the centrality of spatiotemporal resource variation in shaping individual movement strategies across diverse species and add to our understanding of the origins of different movement strategies by encompassing highly local variation among individuals. Our work also reiterates the importance of winter conditions for the movements of temperate ungulates. These findings provide a starting point for more behaviourally informed conservation strategies of free-ranging wildlife. Mapping spatiotemporal variation in resources across diverse landscapes will enable more robust assessment of where different movement strategies are most likely to be found, where they may have already been lost, and how best to conserve each strategy for long-term resiliency of migratory ungulate populations.

## Data Availability

Data and code to recreate the analyses are available on Dryad [78]. Supplementary material is available online [79].

## References

[1] Mueller T, Fagan WF. 2008 Search and navigation in dynamic environments—from individual behaviors to population distributions. Oikos **117**, 654–664. (10.1111/j.0030-1299.2008.16291.x)

[2] Teitelbaum CS, Mueller T. 2019 Beyond migration: causes and consequences of nomadic animal movements. Trends Ecol. Evol. **34**, 569–581. (10.1016/j.tree.2019.02.005) 30885413

[3] Gehrt SD, Anchor C, White LA. 2009 Home range and landscape use of coyotes in a metropolitan landscape: conflict or coexistence? J. Mammal. **90**, 1045–1057. (10.1644/08-mamm-a-277.1)

[4] Schummer ML, Coluccy JM, Mitchell M, Van Den Elsen L. 2017 Long‐term trends in weather severity indices for dabbling ducks in eastern North America. Wildl. Soc. Bull. **41**, 615–623. (10.1002/wsb.837)

[5] Casale P, Freggi D, Basso R, Vallini C, Argano R. 2007 A model of area fidelity, nomadism, and distribution patterns of loggerhead sea turtles (Caretta caretta) in the Mediterranean Sea. Mar. Biol. **152**, 1039–1049. (10.1007/s00227-007-0752-7)

[6] Bauer S, Hoye BJ. 2014 Migratory animals couple biodiversity and ecosystem functioning worldwide. Science **344**, 1242552. (10.1126/science.1242552) 24700862

[7] Kauffman BMJ *et al*. 2021 Mapping out a future for ungulate migrations. Science **372**, 566–569. (10.1126/science.abf0998) 33958460

[8] Nandintsetseg D *et al*. 2019 Challenges in the conservation of wide‐ranging nomadic species. J. Appl. Ecol. **56**, 1916–1926. (10.1111/1365-2664.13380)

[9] Stephens DW, Krebs J. 1986 Foraging theory. Princeton, NJ: Princeton University Press.

[10] Milton K. 1981 Distribution patterns of tropical plant foods as an evolutionary stimulus to primate mental development. Am. Anthropol. **83**, 534–548. (10.1525/AA.1981.83.3.02A00020)

[11] Sawyer H, Lindzey F, McWhirter D. 2005 Mule deer and pronghorn migration in western Wyoming. Wildl. Soc. Bull. **33**, 1266–1273. (10.2193/0091-7648(2005)33[1266:MDAPMI]2.0.CO;2)

[12] Middleton AD *et al*. 2020 Conserving transboundary ungulate migrations: emerging insights and case studies from the greater Yellowstone ecosystem. Front. Ecol. Evol. **18**, 83–91. (10.1002/fee.2145)

[13] Aikens EO *et al*. 2020 Wave-like patterns of plant phenology determine ungulate movement tactics. Curr. Biol. **30**, 3444–3449.(10.1016/j.cub.2020.06.032) 32619482

[14] Peters W *et al*. 2017 Migration in geographic and ecological space by a large herbivore. Ecol. Monogr. **87**, 297–320. (10.1002/ecm.1250)

[15] Pedler RD, Ribot RFH, Bennett ATD. 2014 Extreme nomadism in desert waterbirds: flights of the banded stilt. Biol. Lett. **10**, 20140547. (10.1098/rsbl.2014.0547) 25319819 PMC4272204

[16] Frederick PC, Bildstein KL, Fleury B, Ogden J. 1996 Conservation of large, nomadic populations of white ibises (Eudocimus albus) in the United States. Conserv. Biol. **10**, 203–216. (10.1046/j.1523-1739.1996.10010203.x)

[17] Alerstam T, Hedenström A, Åkesson S. 2003 Long‐distance migration: evolution and determinants. Oikos **103**, 247–260. (10.1034/j.1600-0706.2003.12559.x)

[18] Eggeman S, Hebblewhite M, Bohm H, Whittington J, Merrill EH. 2016 Behavioural flexibility in migratory behaviour in a long‐lived large herbivore. J. Anim. Ecol. **85**, 785–797. (10.1111/1365-2656.12495) 26790111

[19] Singh NJ, Börger L, Dettki H, Bunnefeld N, Ericsson G. 2012 From migration to nomadism: movement variability in a northern ungulate across its latitudinal range. Ecol. Appl. **22**, 2007–2020. (10.1890/12-0245.1) 23210316

[20] Hegemann A, Marra PP, Tieleman BI. 2015 Causes and consequences of partial migration in a passerine bird. Am. Nat. **186**, 531–546. (10.1086/682667) 26655576

[21] Chapman BB, Brönmark C, Nilsson JA, Hansson LA. 2011 The ecology and evolution of partial migration. Oikos **120**, 1764–1775. (10.1111/j.1600-0706.2011.20131.x)

[22] Lundberg P. 1988 The evolution of partial migration in birds. Trends Ecol. Evol. **3**, 172–175. (10.1016/0169-5347(88)90035-3) 21227194

[23] Berg JE, Hebblewhite M, St. Clair CC, Merrill EH. 2019 Prevalence and mechanisms of partial migration in ungulates. Front. Ecol. Evol. **7**, 325. (10.3389/fevo.2019.00325)

[24] Chapman BB, Hulthén K, Brodersen J, Nilsson PA, Skov C, Hansson L-A, Brönmark C. 2012 Partial migration in fishes: causes and consequences. J. Fish Biol. **81**, 456–478. (10.1111/j.1095-8649.2012.03342.x) 22803720

[25] Zuckerman GR *et al*. 2023 Diverse migratory portfolios drive inter‐annual switching behavior of elk across the greater Yellowstone ecosystem. Ecosphere **14**, e4502. (10.1002/ecs2.4502)

[26] Xu W *et al*. 2021 The plasticity of ungulate migration in a changing world. Ecology **102**, e03293. (10.1002/ecy.3293) 33554353

[27] Lundberg P. 1987 Partial bird migration and evolutionarily stable strategies. J. Theor. Biol. **125**, 351–360. (10.1016/S0022-5193(87)80067-X)

[28] Kaitala A, Kaitala V, Lundberg P. 1993 A theory of partial migration. Am. Nat. **142**, 59–81. (10.1086/285529)

[29] Jesmer BR *et al*. 2018 Is ungulate migration culturally transmitted? Evidence of social learning from translocated animals. Science **361**, 1023–1025. (10.1126/science.aat0985) 30190405

[30] Hebblewhite M, Merrill EH. 2009 Trade‐offs between predation risk and forage differ between migrant strategies in a migratory ungulate. Ecology **90**, 3445–3454. (10.1890/08-2090.1) 20120812

[31] Sweeney JM, Sweeney JR. 1984 Snow depths influencing winter movements of elk. J. Mammal. **65**, 524–526. (10.2307/1381113)

[32] Jakes AF, Gates CC, DeCesare NJ, Jones PF, Goldberg JF, Kunkel KE, Hebblewhite M. 2018 Classifying the migration behaviors of pronghorn on their northern range. J. Wildl. Manag. **82**, 1229–1242. (10.1002/jwmg.21485)

[33] Rickbeil GJM *et al*. 2019 Plasticity in elk migration timing is a response to changing environmental conditions. Glob. Chang. Biol. **25**, 2368–2381. (10.1111/gcb.14629) 30908766

[34] Robb BS, Merkle JA, Sawyer H, Beck JL, Kauffman MJ. 2022 Nowhere to run: semi‐permeable barriers affect pronghorn space use. J. Wildl. Manag. **86**, e22212. (10.1002/jwmg.22212)

[35] Sandoval Lambert M, Sawyer H, Merkle JA. 2022 Responses to natural gas development differ by season for two migratory ungulates. Ecol. Appl. **32**, e2652. (10.1002/eap.2652) 35543078 PMC13425686

[36] Mumme S *et al*. 2023 Wherever I may roam—human activity alters movements of red deer (Cervus elaphus) and elk (Cervus canadensis) across two continents. Glob. Chang. Biol. **29**, 5788–5801. (10.1111/gcb.16769) 37306048

[37] Gigliotti LC *et al*. 2023 Multi‐level thresholds of residential and agricultural land use for elk avoidance across the greater Yellowstone ecosystem. J. Appl. Ecol. **60**, 1089–1099. (10.1111/1365-2664.14401)

[38] Sawyer H, Beckmann JP, Seidler RG, Berger J. 2019 Long‐term effects of energy development on winter distribution and residency of pronghorn in the greater Yellowstone ecosystem. Conserv. Sci. Pract. **1**, e83. (10.1111/csp2.83)

[39] Hennig JD, Scasta JD, Pratt AC, Wanner CP, Beck JL. 2023 Habitat selection and space use overlap between feral horses, pronghorn, and greater sage‐grouse in cold arid steppe. J. Wildl. Manage. **87**, e22329. (10.1002/jwmg.22329)

[40] Reinking AK, Smith KT, Mong TW, Read MJ, Beck JL. 2019 Across scales, pronghorn select sagebrush, avoid fences, and show negative responses to anthropogenic features in winter. Ecosphere **10**, e02722. (10.1002/ecs2.2722)

[41] Nelson AA, Kauffman MJ, Middleton AD, Jimenez MD, McWhirter DE, Barber J, Gerow K. 2012 Elk migration patterns and human activity influence wolf habitat use in the greater Yellowstone ecosystem. Ecol. Appl. **22**, 2293–2307. (10.1890/11-1829.1) 23387126

[42] Jones JD, Kauffman MJ, Monteith KL, Scurlock BM, Albeke SE, Cross PC. 2014 Supplemental feeding alters migration of a temperate ungulate. Ecol. Appl. **24**, 1769–1779. (10.1890/13-2092.1) 29210236

[43] Buchanan CB, Wulff SS, Albeke SE, Beck JL. 2023 Elk shift resource selection temporally in response to natural gas development. Rangel. Ecol. Manag. **90**, 35–44. (10.1016/j.rama.2023.05.006)

[44] Brunet MJ, Monteith KL, Huggler KS, Thompson DJ, Burke PW, Zornes M, Lionberger P, Valdez M, Holbrook JD. 2023 Spatiotemporal predictions of the alternative prey hypothesis: predator habitat use during decreasing prey abundance. Ecosphere **14**, e4370. (10.1002/ecs2.4370)

[45] Sikes RS, Animal Care and Use Committee of the American Society of Mammalogists. 2016 2016 Guidelines of the American Society of Mammalogists for the use of wild mammals in research and education. J. Mammal. **97**, 663–688. (10.1093/jmammal/gyw078) 29692469 PMC5909806

[46] Bunnefeld N, Börger L, van Moorter B, Rolandsen CM, Dettki H, Solberg EJ, Ericsson G. 2011 A model-driven approach to quantify migration patterns: individual, regional and yearly differences. J. Anim. Ecol. **80**, 466–476. (10.1111/j.1365-2656.2010.01776.x) 21105872

[47] Cagnacci F *et al*. 2016 How many routes lead to migration? Comparison of methods to assess and characterize migratory movements. J. Anim. Ecol. **85**, 54–68. (10.1111/1365-2656.12449) 26412564

[48] Nielson RM, Sawyer H, McDonald TL. 2013 BBMM: Brownian bridge movement model version 3.0.

[49] R Core Team. 2020 R: a language and environment for statistical computing. Vienna, Austria: R Foundation for Statistical Computing.

[50] Fieberg J, Kochanny CO. 2005 Quantifying home-range overlap: the importance of the utilization distribution. J. Wildl. Manag. **69**, 1346. (10.2193/0022-541X(2005)69)

[51] Abrahms B, Hazen EL, Bograd SJ, Brashares JS, Robinson PW, Scales KL, Crocker DE, Costa DP. 2018 Climate mediates the success of migration strategies in a marine predator. Ecol. Lett. **21**, 63–71. (10.1111/ele.12871) 29096419

[52] Calenge C. 2015 Home range estimation in R: the adehabitatHR Package. R Vignette 1–60. https://github.com/clementcalenge/adehabitathr

[53] Lowrey B, McWhirter DE, Proffitt KM, Monteith KL, Courtemanch AB, White PJ, Paterson JT, Dewey SR, Garrott RA. 2020 Individual variation creates diverse migratory portfolios in native populations of a mountain ungulate. Ecol. Appl. **30**, eap.2106. (10.1002/eap.2106) 32091631

[54] van de Kerk M, Larsen RT, Olson DD, Hersey KR, McMillan BR. 2021 Variation in movement patterns of mule deer: have we oversimplified migration? Mov. Ecol. **9**, 44. (10.1186/s40462-021-00281-7) 34446100 PMC8394567

[55] Couriot O *et al*. 2018 Truly sedentary? The multi-range tactic as a response to resource heterogeneity and unpredictability in a large herbivore. Oecologia **187**, 47–60. (10.1007/s00442-018-4131-5) 29610976

[56] Merkle JA *et al*. 2016 Large herbivores surf waves of green-up during spring. Proc. R. Soc. B **283**, 20160456. (10.1098/rspb.2016.0456) PMC4936031 27335416

[57] Aikens EO, Kauffman MJ, Merkle JA, Dwinnell SPH, Fralick GL, Monteith KL. 2017 The greenscape shapes surfing of resource waves in a large migratory herbivore. Ecol. Lett. **20**, 741–750. (10.1111/ele.12772) 28444870

[58] Middleton AD, Merkle JA, McWhirter DE, Cook JG, Cook RC, White PJ, Kauffman MJ. 2018 Green‐wave surfing increases fat gain in a migratory ungulate. Oikos **127**, 1060–1068. (10.1111/oik.05227)

[59] Abrahms B, Aikens EO, Armstrong JB, Deacy WW, Kauffman MJ, Merkle JA. 2021 Emerging perspectives on resource tracking and animal movement ecology. Trends Ecol. Evol. **36**, 2020. (10.1016/j.tree.2020.10.018) 33229137

[60] Proffitt KM, Terrill Paterson J, DeVoe JD, Hansen CP, Millspaugh JJ. 2025 Evaluating patterns of plant phenological progression and pronghorn movement behaviors across diverse landscapes. Wildl. Monogr. **218**, e70003. (10.1002/wmon.70003)

[61] Poole KG, Lamb CT, Medcalf S, Amos L. 2024 Migration, movements, and survival in a partially migratory elk (Cervus canadensis) population. Conserv. Sci. Pract. **6**, e13128. (10.1111/csp2.13128)

[62] Hebblewhite M, Merrill EH. 2011 Demographic balancing of migrant and resident elk in a partially migratory population through forage–predation tradeoffs. Oikos **120**, 1860–1870. (10.1111/j.1600-0706.2011.19436.x)

[63] Martin HW, Hebblewhite M, Merrill EH. 2022 Large herbivores in a partially migratory population search for the ideal free home. Ecology **103**, e3652. (10.1002/ecy.3652) 35084736 PMC10162400

[64] Paterson JT, Johnston AN, Ortega AC, Wallace C, Kauffman M. 2023 Hidden Markov movement models reveal diverse seasonal movement patterns in two North American ungulates. Ecol. Evol. **13**, e10282. (10.1002/ece3.10282) 37484933 PMC10361361

[65] Gigliotti LC *et al*. 2022 Wildlife migrations highlight importance of both private lands and protected areas in the greater Yellowstone ecosystem. Biol. Conserv. **275**, 109752. (10.1016/j.biocon.2022.109752)

[66] Cagnacci F *et al*. 2011 Partial migration in roe deer: migratory and resident tactics are end points of a behavioural gradient determined by ecological factors. Oikos **120**, 1790–1802. (10.1111/j.1600-0706.2011.19441.x)

[67] Sabine DL, Morrison SF, Whitlaw HA, Ballard WB, Forbes GJ, Bowman J. 2002 Migration behavior of white-tailed deer under varying winter climate regimes in New Brunswick. J. Wildl. Manage. **66**, 718. (10.2307/3803137)

[68] LaSharr TN *et al*. 2023 Behavior, nutrition, and environment drive survival of a large herbivore in the face of extreme winter conditions. Ecosphere **14**, e4601. (10.1002/ecs2.4601)

[69] Abrahms B, Aikens EO, Armstrong JB, Deacy WW, Kauffman MJ, Merkle JA. 2021 Emerging perspectives on resource tracking and animal movement ecology. Trends Ecol. Evol. **36**, 308–320. (10.1016/j.tree.2020.10.018) 33229137

[70] Xu W, Dejid N, Herrmann V, Sawyer H, Middleton AD. 2021 Barrier behaviour analysis (BaBA) reveals extensive effects of fencing on wide‐ranging ungulates. J. Appl. Ecol. **58**, 690–698. (10.1111/1365-2664.13806)

[71] Strandburg-Peshkin A, Farine DR, Couzin ID, Crofoot MC. 2015 Shared decision-making drives collective movement in wild baboons. Science **348**, 1358–1361. (10.1126/science.aaa5099) 26089514 PMC4801504

[72] Sigaud M, Merkle JA, Cherry SG, Fryxell JM, Berdahl A, Fortin D. 2017 Collective decision‐making promotes fitness loss in a fusion‐fission society. Ecol. Lett. **20**, 33–40. (10.1111/ele.12698) 27873440

[73] Papageorgiou D, Farine DR. 2020 Shared decision-making allows subordinates to lead when dominants monopolize resources. Sci. Adv. **6**, 5881–5906. (10.1126/sciadv.aba5881) PMC7688327 33239284

[74] Rivrud IM, Bischof R, Meisingset EL, Zimmermann B, Loe LE, Mysterud A. 2016 Leave before it’s too late: anthropogenic and environmental triggers of autumn migration in a hunted ungulate population. Ecology **97**, 1058–1068. (10.1890/15-1191.1) 28792596

[75] Bolger DT, Newmark WD, Morrison TA, Doak DF. 2008 The need for integrative approaches to understand and conserve migratory ungulates. Ecol. Lett. **11**, 63–77. (10.1111/j.1461-0248.2007.01109.x) 17897327

[76] Dobson AP *et al*. 2010 Road will ruin Serengeti. Nature **467**, 272–273. (10.1038/467272a) 20844519

[77] Berger J, Cain SL. 2014 Moving beyond science to protect a mammalian migration corridor. Conserv. Biol. **28**, 1142–1150. (10.1111/cobi.12327) 24962197

[78] Becker JA *et al*. 2025 The role of spatiotemporal variation in resources in the diverse movement strategies of temperate ungulates. Dryad Digital Repository. (10.5061/dryad.mgqnk998d)41290171

[79] Becker JA *et al*. 2025 Supplementary material from: The role of spatiotemporal variation in resources in the diverse movement strategies of temperate ungulates. Figshare. (10.6084/m9.figshare.c.8156185)41290171

